# Bacterial Endotoxin Testing—Fast Endotoxin Masking Kinetics in the Presence of Lauryldimethylamine Oxide

**DOI:** 10.3390/microorganisms8111728

**Published:** 2020-11-04

**Authors:** René Bech Ørving, Bill Carpenter, Steffen Roth, Johannes Reich, Birgitte H. Kallipolitis, Jacob Sonne-Hansen

**Affiliations:** 1 FUJIFILM Diosynth Biotechnologies, 3400 Hillerød, Denmark; REO@novonordisk.com; 2 Biogen, Cambridge, MA 02142, USA; bill.carpenter@biogen.com; 3 Microcoat Biotechnologie GmbH, 82347 Bernried, Germany; s.roth@microcoat.de (S.R.); j.reich@microcoat.de (J.R.); 4 Department of Biochemistry and Molecular Biology, University of Southern Denmark, 5230 Odense, Denmark; bhk@bmb.sdu.dk

**Keywords:** low endotoxin recovery, LER, masking, limulus amoebocyte lysate, LAL, endotoxin, LPS, lauryldimethylamine oxide, LDAO

## Abstract

For release of parenteral drug products, bacterial endotoxin testing is one of a panel of necessary tests. In order to ensure the validity of such tests, various controls are performed, including demonstration of compendial method suitability or method qualification. In addition to compendial suitability testing, quality control (QC) sample hold-time studies are requested by authorities like the Food and Drug Administration (FDA) as described in “Guidance for Industry: Pyrogen and Endotoxins Testing.” It is requested to be determine whether the ability to detect endotoxins can be affected by storage and handling of the sample to be tested. To accomplish these studies, endotoxin is introduced or spiked into the undiluted product and held for a certain period of time in process-representative containers. This time period reflects procedural maximum QC sample hold time from sampling until analysis. Inadequate detection of endotoxin can be caused by adsorption of endotoxin to container surfaces or molecular masking effects, in which the binding sites on the endotoxin molecules are prevented from triggering the enzymatic cascade necessary in the assay, are obscured. The endotoxin may form macromolecular structures, such as sheets or blebs, or the binding sites may otherwise be rendered unavailable due to the sample matrix composition. In either case, the endotoxin assay may yield falsely low results if and when masking occurs. In this work, the QC sample hold times of different in-process controls within the production process of a biopharmaceutical product were analyzed. One out of eight different samples showed a strong masking of endotoxin. Analysis of the sample composition revealed that either kifunensine, mycophenolic acid (MPA), or lauryl-N, N-dimethylamine oxide (LDAO) was responsible for masking. Further analysis clearly identified LDAO as the root cause for masking. A novel one-step mechanism for LDAO-induced endotoxin masking is proposed. The principle is similar to an already-proposed two-step mechanism for endotoxin masking, but the LDAO case combines these two steps: the disturbance of the salt bridges and hydrophobic interactions with LPS in one molecule. These molecular interactions occur quickly when both endotoxin and LDAO are present in the same matrix. Thus, depending on the masking agents, low endotoxin recovery (LER) can occur regardless of the QC sample hold duration.

## 1. Introduction

Bacterial endotoxins are a group of lipopolysaccharides (LPS) found in Gram-negative bacteria. As part of the outer membrane, endotoxins are involved in regulatory mechanisms for cell viability, such as nutrient uptake, but are also essential for the protection of the cell from its surrounding environment [1,2]. Structurally, endotoxins can be divided into three regions: lipid A, core region, and O-chain. Lipid A is a glycophospholipid harboring hydrophobic regions due to fatty acid residues, but also ionic regions through phosphate groups in sugar residues [2,3]. Both regions are important for the linkage of endotoxin molecules to the outer membrane. The hydrophobic region interacts with the acyl residues of the outer membrane, whereas the connection is further stabilized by the formation of salt bridges between the phosphate groups of neighboring lipid A molecules and divalent cations. The core region consists of different conserved sugar residues and is highly negatively charged due to various carboxyl and phosphate groups, which further enhance the binding between endotoxin molecules via salt bridges. The O-chain consists of different repeating oligosaccharide units and is the most exposed region of the endotoxin to environmental factors [2,3]. The most conserved part of LPS in the outer membrane of bacteria is lipid A, which is known as a strong stimulator of the natural and innate immune system in eukaryotic species. In humans, fever and organ failure can result from endotoxin presence in the circulatory system [4,5]. Thus, the detection of endotoxin is of utmost importance, especially for parenteral drugs, both in drug manufacturing process intermediate samples and in the final parenteral drug product [6].

Historically speaking, the earliest endotoxin test was the rabbit pyrogen test (RPT). Briefly, in this assay, samples are injected into rabbits, and the increase of the body temperature is measured [7]. Besides endotoxin, other pyrogens (i.e., non-endotoxin pyrogens) are detected by this test [8]. For a higher specificity for endotoxin detection, the RPT has largely been replaced by limulus amoebocyte lysate (LAL)-based assays [9,10]. All LAL assays detect endotoxin activities and are based on the endotoxin-specific activation of factor C, the first enzyme within the blood coagulation cascade of horseshoe crabs. The cascade further includes factor B, proclotting enzyme, and others. Based on this cascade, different assay designs are available, and endotoxin can be quantified by either gel formation, turbidity, or release of a chromogenic substrate. One of the drawbacks of LAL is its cross-induction by beta-glucans. Via a side pathway, the cascade can be activated by factor G in the presence of beta-glucans [11]. 

Driven by the drawbacks of the LAL assays, alternative methods for endotoxin detection utilizing recombinant factor C (rFC) have been established [12,13,14]. As in the LAL-based assays, factor C from a recombinant source is activated by LPS. In contrast to LAL-based assays, activated factor C directly reacts with a fluorogenic substrate. Compared with LAL-based assays, rFC assays are highly endotoxin-specific due to the elimination of all additional horseshoe crab blood constituents. Moreover, rFC can be produced in a sustainable manner without the use of animals with the additional benefit of a decreased interlot variability. Recent studies have shown that any concerns about using rFC-based methods instead of LAL-based methods for endotoxin detection are comparable [13,14,15]. Overall, a comparable determination of endotoxin activities was possible with both methods, rFC and LAL-based assays. In addition, there is another test available for the detection of pyrogens: the so-called monocyte activation test (MAT). This method detects endotoxin and non-endotoxin pyrogens [16]. The principle of the test is based on the activation of human monocytes by different pyrogenic substances triggering the release of cytokines, such as IL-1β, TNF-α, and IL-6, which are quantified by ELISA. Therefore, the assay is not specific for endotoxin, but broadly sensitive for many pyrogenic substances [17]. Compared with the RPT, the MAT is a non-animal-based method and provides advantages in terms of sensitivity. The MAT is intended to replace the RPT. 

Although various methods for endotoxin detection with high sensitivities are available, in 2013, Chen and Vinther reported that they were unable to recover a defined amount of endotoxin when spiked into samples and held for a certain period of time [18]. This phenomenon is called low endotoxin recovery (LER) and is caused by the masking of endotoxin. This finding showed that in order to investigate whether endotoxin masking occurs in a given matrix, recovery studies need to be performed. Therefore, undiluted samples are spiked with endotoxin and incubated for a certain period of time. In pharmaceutical industries, there are two scenarios in which such endotoxin recovery studies are of interest. In both scenarios, the undiluted sample is spiked with endotoxin, but incubation temperatures and hold time vary.

Scenario 1 is the determination of whether or not a sample shows masking under conditions that are relevant for the manufacturing process. Such studies are called LER hold-time studies, and detailed information is provided, for example, in Parenteral Drug Association (PDA) Technical Report No. 82 [19]. The conditions for an LER sample hold-time study are chosen dependent on the manufacturing process. For example, if the product is processed at room temperature, the LER hold-time study should be performed at room temperature too. The period of time relates to the manufacturing process. Due to the relatively fast molecular kinetics at room temperature [20,21], hold times up to 7 days should be sufficient to identify a masking effect.

Scenario 2 is the determination of whether or not a sample shows masking under conditions that are relevant for QC sample storage. These studies are called QC sample hold-time studies and, based on question 3 in FDA’s Guidance for Industry, determine if sample storage and handling is important for pyrogen and endotoxin recovery accuracy [22]. For example, the hold time for QC samples corresponds to generic time periods, which are experienced from the time point of sampling until analysis of the sample (i.e., 14 days). The incubation temperature in the study should correspond to the storage temperature (i.e., samples are stored in the fridge at 2–8 °C). From a mechanistic point of view, both scenarios can reveal the same driving forces of endotoxin masking. In some cases, reduced incubation temperature may only suppress or decelerate the mechanisms responsible for the masking of endotoxin; thus hold times longer than 7 days can be relevant.

The different scenarios are described in order to assist the reader with choosing the right hold time and temperature for a study to validate a bacterial endotoxin test method and desired sample hold time. The following work was intended to investigate QC sample hold-time studies. Notably, endotoxin masking has to be clearly differentiated from test method interference. Most test interferences are matrix concentration dependent and can be overcome by sample dilution [23]. The influence of the sample on the assay is analyzed by spiking a defined amount of endotoxin shortly before measurement into the diluted sample (positive product control or PPC). The test is then considered to be valid if the PPC recoveries are in the range of 50%–200% of the theoretical spike.

In contrast, to investigate masking effects, endotoxin is spiked into the undiluted sample and analyzed at qualified dilutions. Endotoxin recoveries below 5% of the nominal or spiked water control value indicate masking effects [19,24]. It is hypothesized that endotoxin masking is driven by the change of the supramolecular structure of the endotoxin [19,21]. Light scattering experiments on LPS under LER conditions support this hypothesis [25]. Furthermore, a two-step process that is induced by different components of the formulation or the active pharmaceutical ingredient (API) itself has been proposed [21,26]. First, the salt bridges between the LPS molecules are destabilized by the interaction of complex-forming agents with divalent cations. In the second step, the supramolecular structure of endotoxin is altered by the formation of mixed aggregates with different formulation components. As a consequence, the activation of factor C of LAL-based tests is hindered. The interaction of formulation components and endotoxin resulting in a nondetectable state of the endotoxin is also called endotoxin masking.

Since 2013, there have been many controversial discussions in the pharmaceutical industry concerning the phenomenon of low endotoxin recovery. There was a debate regarding purified and nonpurified endotoxin. It was postulated that the LER effect is only triggered by purified endotoxin (LPS) rather than by sections of Gram-negative cell membranes as they are distributed naturally in matrices as tested. Obviously, most bacterial endotoxin test experiments are performed with endotoxin standards, which are highly purified. In some cases, in which LPS had shown LER in a given matrix, no LER was observed when crude endotoxin extracts (also called NOE) were used. However, it could not be proven that the improved recovery was solely due to the crude endotoxin preparation (i.e., less purity); it has rather been shown that the susceptibility of endotoxin masking is dependent on the bacterial species and conditions of growth [27]. The latter may result in structural modifications, like the degree of acylation; substitution with, for example, aminoethanol; and variation in the polysaccharide (i.e., O-antigen). As a consequence, to sensitively investigate whether a sample of interest is prone to endotoxin masking, an endotoxin that is highly susceptible (i.e., reference or control standard endotoxins) to masking must be used. 

Some product and formulation combinations (e.g., positively charged proteins, chelators, and detergents) have been shown to lead to the formation of a macromolecular complex, and thus to the masking of the endotoxin. As a result, this could lead to false negative results or underestimated endotoxin contaminations, which in turn is a potential risk to patients [28]. Dedicated endotoxin spiking experiments are performed to investigate whether a drug product is prone to endotoxin masking under relevant production and storage conditions. In the present work, QC sample hold-time studies on different process steps during biopharmaceutical drug product manufacturing were conducted, and one new component, LDAO, that causes strong endotoxin masking by a one-step mechanism has been identified. 

## 2. Materials and Methods

LAL kinetic turbidimetric test, reconstitution buffer, LAL reagent water (LRW), control standard endotoxin from *E.coli* O55:B5 (CSE, 100 EU/mL), vials, and N201 test tubes with polypropylene screw caps were obtained from Lonza, Copenhagen, Denmark. Kifunensine was obtained from GlycoSyn Technologies, Lower Hutt, New Zealand. Mycophenolic acid (MPA) was obtained from Chongqing Daxin Pharmaceutical Co., Chongqing, China, and lauryl-N, N-dimethylamine oxide (LDAO) was obtained from SAFC/Sigma-Aldrich, Buchs, Switzerland. All material for consumption was certified pyrogen-free.

The analyzed samples were derived from downstream manufacturing steps of a biopharmaceutical drug product process and reflect selected steps of in-process controls routinely tested. An overview of the individual steps and components is given in Table 1 below.

A BioTek ELx808 Ultra Microplate Reader measuring light absorbance at 340 nm was obtained from Biotek, Hillerød, Denmark. For control and interpretation of endotoxin measurements, WinKQCL (Version 6.0) Software from Lonza, Copenhagen, Denmark, was used. E-pipettes with volumes of 100, 300, and 1000 µL were obtained from Biohit Automatic, Hillerød, Denmark. For sample vortexing, the device IKA-Vibrax VXR basic, Hillerød, Denmark, and a Fisherbrand mini mixer from Fisher Scientific, Hillerød, Denmark, were used.

Endotoxin detection was performed using the kinetic turbidimetric LAL assay. The test was performed according to the manufacturer’s instructions. A minimum 4-point curve using standards of 0.01, 0.1, 1.0, and 10 EU/mL was used. Qualified sample dilutions were determined according to the European Pharmacopoeia, chapter 2.6.14 [29].

### 2.1. Study Design

In order to spike undiluted samples, the reverse mode according to Technical Report No. 82 [19] was chosen for the study design. Thus, samples were prepared by spiking a defined amount of endotoxin into the samples at a specific time point, starting with the latest samples (day 10, maximum hold time). Additional samples were prepared the same way and held for shorter periods ending with time point zero samples, which were directly analyzed after spiking (*T*_0_ = 0 h). A water control was included by spiking LRW with the same amount of endotoxin at each time point and was incubated in parallel. A final concentration of 10 EU/mL in the samples and water controls was achieved by adding 200 µL Control Standard Endotoxin (CSE) stock solution (100 EU/mL) to 1800 µL product or LRW, respectively. All samples were stored at 2–8 °C until measurement. Using the reverse mode, all samples were analyzed at the same time on one assay, thereby eliminating interassay variability.

### 2.2. Calculation of Recovery

Recovery of endotoxin in each sample at a given time point is calculated against the measured endotoxin value of the LRW control at time point zero (*T*_0_) and given in percent.

$$\mathrm{Recovery}\;\left(\mathrm{sample}\right)\;=\;\frac{\mathrm{Measured}\;\mathrm{value}\;\mathrm{sample}\;\left(\frac{EU}{mL}\right)}{\mathrm{Measured}\;\mathrm{value}\;\mathrm{water}\;\mathrm{control}\;T0\;\left(\frac{EU}{mL}\right)}\times \;100$$

Recoveries less than 50% in two consecutive time points of the expected value indicate a LER effect.

## 3. Results

**Figure 1:**
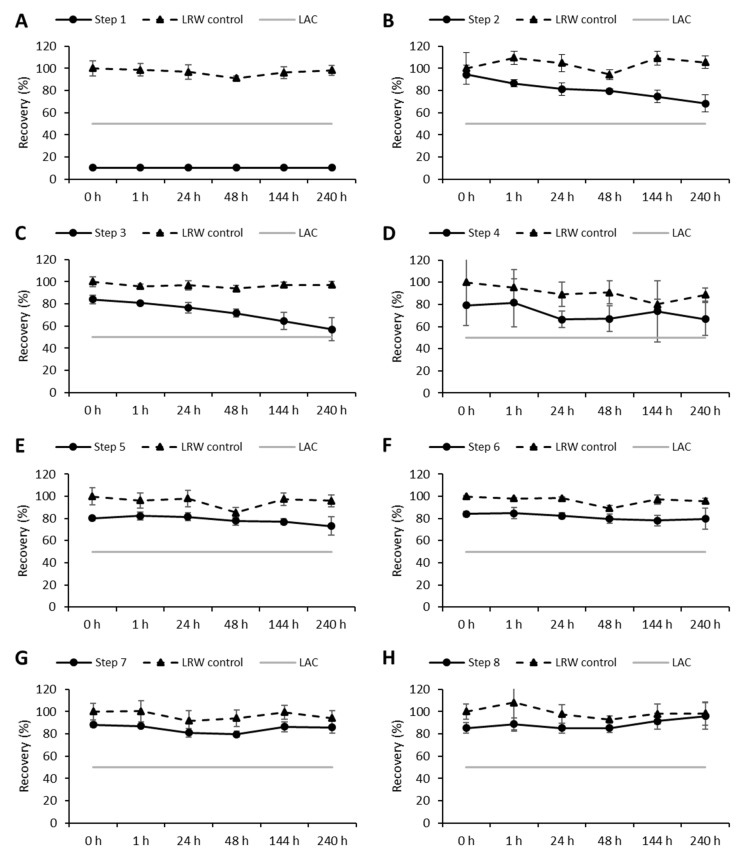
Results of the hold-time study of eight in-process steps. Endotoxin recoveries for each step and corresponding LRW controls are shown. The initial spike was set to 10 EU/mL, followed by an incubation up to 10 days at 2 to 8 °C. For reasons of graphical clarity, recoveries are represented as mean of three independent sample lots and water controls, respectively. Error bars represent standard deviation of three sample lots. Time point zero (*T*_0_) of the LRW control was used as reference value for calculations. LAC: lower acceptance criteria for endotoxin recovery (50% endotoxin recovery); (**A**) step 1: virus inactivation; (**B**) step 2: affinity chromatography; (**C**) step 3: pH adjustment; (**D**) step 4: anion exchange; (**E**) step 5: cation exchange; (**F**) step 6: virus filtration; (**G**) step 7: ultrafiltration; (**H**) step 8: drug substance formulation. *Y*-axis: endotoxin recovery referred to LRW control *T*_0_ (%); *x*-axis: different time points.

The manufacturing process for biopharmaceutical components is normally divided into upstream and downstream processes. Especially for the downstream process, several steps are necessary in order to provide consistent pharmaceutical agents at high purities. For example, anion exchange chromatography can be used to remove endotoxin from therapeutic proteins [30]. These process steps often require matrix changes due to the addition of buffers and water and are also subject to contamination as prefiltration may not remove the endotoxin. The recovery of endotoxin in the assay can be affected by both the amount of endotoxin present and the innate interference in recovery that the matrix shows. On the one hand, at each step, the sample could be contaminated with endotoxin by the addition of or contact with other solutions. On the other hand, the matrix itself directly influences the recovery of endotoxin in bacterial endotoxin tests. For that reason, eight different downstream process steps (virus inactivation, affinity chromatography, pH adjustment, anion exchange, cation exchange, virus filtration, ultrafiltration, and drug substance formulation) within the industrial production of a biopharmaceutical agent were analyzed regarding their endotoxin content and endotoxin masking capability. In Figure 1, the recoveries of each in-process step and the corresponding LRW controls over time are shown. Three individual lots of each process step were analyzed. 

Per definition, the masking of endotoxin is present in a sample if the recoveries of spiked endotoxin at two consecutive time points are below 50%. In this study, none of the samples from steps 2 to 8 fulfilled the criterion for endotoxin masking or LER during the analyzed time period (Figure 1B–H). However, for in-process step 1, endotoxin recoveries were below 50% at time point zero (*T*_0_) and every other following time point (below limit of detection (LOD), < 10%), indicating a strong masking capability of the sample. Interestingly, the sample composition of step 1 is similar to the one of step 2, with the exception of three ingredients: (i) mycophenolic acid (MPA), (ii) kifunensine, and (iii) lauryl-N, N-dimethylamine oxide (LDAO). Consequently, the masking effect of each substance was analyzed in more detail.

**Figure 2:**
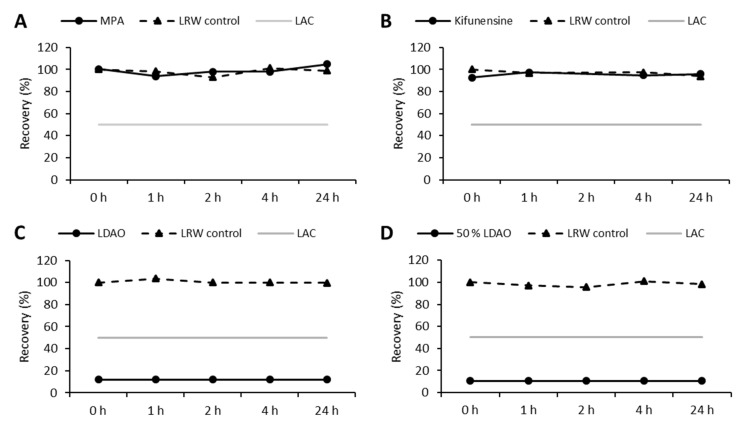
Masking capability of MPA, kifunensine, and LDAO in LRW. Concentration of each component was equal to process conditions. Endotoxin recoveries for each step and corresponding water controls are shown. The initial spike was set to 10 EU/mL, followed by incubation for 24 h at 2 to 8 °C. LAC: lower acceptance criteria for endotoxin recovery (50% endotoxin recovery); (**A**) MPA in LRW at process concentration; (**B**) kifunensine in LRW at process concentration; (**C**) LDAO in LRW at process concentration; (**D**) LDAO in LRW at 50% process concentration. *Y*-axis: endotoxin recovery referred to water control *T*_0_ (%); *x*-axis: different time points.

In a second independent hold time study, the masking capability of MPA, kifunensine, and LDAO was tested separately. The previous experiment revealed masking of endotoxin at *T*_0_; thus the incubation time for this study was reduced to 24 h (Figure 2A–D).

Endotoxin recoveries for both MPA and kifunensine were above 50% for all analyzed time points and similar to the LRW controls (93% to 105%, Figure 2A,B). In contrast, endotoxin recoveries clearly below 50% were observed if LDAO was present in the sample (below LOD, < 12%, Figure 2C). Even lowering the concentration of LDAO did not improve endotoxin recovery (below LOD, < 11%, Figure 2D). These results indicate that neither kifunensine nor MPA is responsible for endotoxin masking in step 1; rather, LDAO is the major reason.

**Figure 3:**
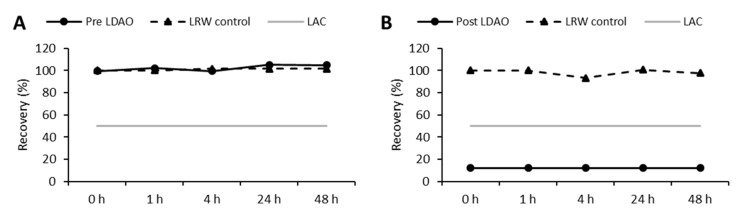
Masking capability of in-process samples directly before and after addition of LDAO. Endotoxin recoveries for each step and corresponding LRW controls are shown. The initial spike was set to 10 EU/mL, followed by incubation for 24 h at 2 to 8 °C. LAC: lower acceptance criteria for endotoxin recovery (50% endotoxin recovery); (**A**) before addition of LDAO; (**B**) after addition of LDAO. *Y*-axis: endotoxin recovery referred to LRW control T_0_ (%); *x*-axis: different time points.

After the strong masking capability of LDAO in LRW was shown, the influence of LDAO under process conditions was further investigated. For that reason, two samples, one directly before (pre-LDAO, Figure 3A) and one directly after (post-LDAO, Figure 3B) LDAO addition, were analyzed in a third hold-time study.

Stable endotoxin recoveries for the sample without LDAO were observed for the complete incubation period (99% to 105%, Figure 3A). In contrast, endotoxin recoveries clearly dropped below 50% in the presence of LDAO (below LOD, < 12%, Figure 3B). The only difference between the two samples was the additional LDAO. Thus, LDAO seems to be the major reason for endotoxin masking in the formulation of process intermediate step 1.

## 4. Discussion

**Figure 4:**
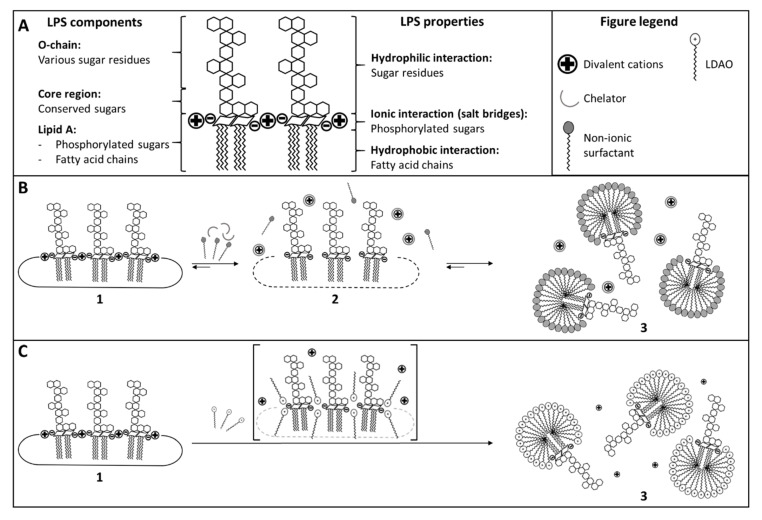
Comparison of potential masking mechanisms (**A**): Simplified schematic structure and resulting properties of endotoxin (LPS). The LPS molecule consists of hydrophilic (due to the sugar residues of the O-chain as well as the core region), ionic (due to the phosphorylated and therefore negatively charged sugars of lipid A), and hydrophobic (due to the fatty acids of lipid A) regions. For that reason, LPS could interact with various molecules via its different regions. (**B**) Masking of endotoxin according to the two-step mechanism. The supramolecular structure of endotoxin in an aqueous environment is dependent on the interaction of its hydrophobic regions and the formation of salt bridges between LPS molecules and divalent cations (state 1). Addition of non-ionic surfactants (i.e., polysorbate 20) and a chelator (i.e., citrate). Rigidity of LPS aggregates is reduced by binding the cations (state 2). Non-ionic surfactants are now able to form mixed aggregates and micelles (state 3). (**C**) Masking of endotoxin by LDAO. Same starting conditions as in A (state 1). Due to the ionic head, LDAO is able to intercalate between the LPS molecules and replace divalent cations. The hydrophobic interaction of LDAO with LPS further improves the intercalation efficiency between LPS molecules and leads to the disruption of the supramolecular structure of LPS. As a result, mixed aggregates can be formed similar to the two-step mechanism, but in a single step (state 3).

The masking capability of different QC hold-time samples during the downstream or purification process of a pharmaceutical drug was analyzed. In total, seven out of eight steps did not show any masking of endotoxin. However, the analysis of the first step revealed a strong masking of endotoxin (Figure 1A). The masking of endotoxin follows a two-step mechanism, which can be caused by the formulation components of a drug product and/or by the API itself. First, a chelator (complex-forming agent) disturbs the salt bridges between LPS molecules and divalent cations by binding the cations (Figure 4B). Thus, the rigidity of the LPS aggregates is reduced, which allows a non-ionic surfactant to interact with the LPS molecules in the second step. In the end, the supramolecular structure of the endotoxin is changed by the formation of mixed aggregates [26,27].

Both a complex-forming agent (phosphate) and a surfactant (LDAO) were also present within the sample, which showed strong endotoxin masking. Thus, the masking of endotoxin according to the reported two-step mechanism may be expected and indeed was observed at the first time point *T*_0_ for step 1 (Figure 1A). Nevertheless, this result is surprising as endotoxin masking, according to the two-step mechanism, is reported to be kinetically controlled and therefore is time-dependent [19,26,27]. Crucial for masking is the complex formation of divalent cations, which is also the limiting step for the reaction kinetics. The reaction kinetic is further influenced by the energy input into the system. For example, Reich et al. could show that the masking of endotoxin is temperature-dependent. The reaction kinetics for the masking of endotoxin increased with increasing temperatures in the range of 2 to 37 °C [26]. In contrast, in this study, very fast masking was observed even at low temperatures (2 to 8 °C, Figure 1A). Compared with the two-step mechanism, the most striking difference in the present study is the extremely accelerated kinetics of endotoxin masking.

Furthermore, a preparation of LDAO in LRW without any additional substances (e.g., buffers or chelators) induced masking (Figure 2). These observations are in disagreement with the previously reported mechanism, as one would assume a matrix would contain two independent moieties to trigger the two-step masking process. The interaction of surfactants with LPS is based on the previous destabilization of the LPS structure by the chelator. In the absence of a chelator, the surfactant should not be able to interact with the LPS structure due to its rigidity. Thus, the masking induced by LDAO may arise from another mechanism. Compared with non-ionic surfactants, which are known to induce endotoxin masking (e.g., polysorbates) in the presence of a chelator, LDAO is an ionic surfactant (Figure 4A).

Structurally, LDAO is a zwitterionic surfactant with a polar amine oxide head and a hydrophobic dodecyl tail. The pH of the step 1 sample is in a low range (pH < 4); thus LDAO is suggested to carry a positive net charge. Additionally, by the disruption of the cell membrane of Gram-negative bacteria, LDAO shows an antimicrobial activity and therefore is highly suspected for interacting with cell membrane structures such as endotoxin [31]. Furthermore, LDAO alone is likely to form micelles above a concentration of 1.70 mM (final concentration in the sample > 1.70 mM). As both features are necessary for the masking of endotoxins, and LDAO displays both of them in one molecule, it appears to be an ideal candidate for the masking of endotoxin. A hypothetical mechanism for the masking effect of LDAO is shown in Figure 4C.

The positively charged head of LDAO may intercalate LPS molecules and attach to the negatively charged lipid A and core region due to ionic interactions. Therefore, the cations of the salt bridges between LPS molecules could be replaced step-by-step by the positively charged head of LDAO. Consequently, the rigidity between the LPS molecules may be reduced. The intercalation could be further stabilized by the hydrophobic tail of LDAO. The C_12_ alkyl chain and the fatty acid residues of lipid A could accumulate via hydrophobic interactions and completely destabilize the LPS aggregates. Compared with the sterically sophisticated polar head of polysorbate 20, LDAO consists of a small amine oxide and a linear C-chain with a similar length as the fatty acids of lipid A. Therefore, the intercalation driven by ionic interactions and the binding towards the fatty acids of lipid A of LDAO may be favored. Additionally, at high concentrations, LDAO is prone to form micelles in aqueous solutions. For that reason, it is likely that LPS and LDAO form mixed aggregates and potentially mixed micelles under these conditions. Thus, the supramolecular structure of the endotoxin may be changed. In such a new conformation, endotoxin may no longer be accessible for factor C and therefore may not be detectable.

From a physicochemical point of view, LDAO combines the same interaction possibilities as the known endotoxin-masking components citrate and polysorbate, but in one molecule. For that reason, LDAO-induced endotoxin masking appears to be a one-step mechanism, but follows the molecular procedure of the two-step mechanism. According to the two-step mechanism, a chelator (disturbance of the salt bridges) and a non-ionic surfactant (formation of mixed aggregates and micelles) are required. Due to the ionic head and the hydrophobic tail, LDAO combines the properties of both substance classes, which are necessary for masking according to the two-step mechanism in one molecule. Thus, the masking of endotoxin is not time-dependent and immediately observed in the presence of LDAO. These results show that endotoxin masking can be triggered by different molecules or combinations thereof and is not limited to typical known masking excipients like polysorbate and citrate. As previously discussed, the time-limiting factor in masking endotoxin is the chelation of divalent cations [26]. Under certain conditions (e.g., nutrient deficiency) bacteria modify their LPS structure by substituting divalent cations with, for example, aminoethanol. In such a case, destabilization of LPS aggregates by chelators is limited. Due to the capability of LDAO to directly interact with LPS molecules, which in turn allows very fast kinetics, it is anticipated that the masking susceptibility of endotoxin is less dependent on modifications of endotoxin. 

With regard to patient safety, the impact of masked endotoxin in a parenteral drug product remains unclear. Under masking conditions, an endotoxin contamination could be underestimated and, subsequently, the drug cleared for patient administration despite its health risk. However, although drug products with typical LER formulations have been on the market for more than 20 years, side effects have not be directly related to endotoxin (masking) yet. This lack of understanding may fuel arguments in favor of the negligence of the phenomenon of endotoxin masking. Nevertheless, fever is commonly observed as an adverse reaction to biopharmaceutical drugs [24,32]. Despite its mechanistic importance, in many cases, there is no investigation as to whether this fever is triggered by the active drug itself or by endotoxin contaminations. In addition to fever, other more severe immunogenic reactions cannot be excluded in the presence of masked endotoxin. Consequently, it is very difficult to elucidate whether masked endotoxin is a concern for patient safety based on available data. Since Schwarz et al. showed that masked LPS is a potent trigger of immune responses, the potential risk of masked LPS was reiterated, as it may pose a health threat in pharmaceutical products or compromise experimental results [33]. Thus, there is general concern from authorities that under certain circumstances, bacterial endotoxin tests lead to an underestimation of an endotoxin contamination. 

In conclusion, the potency for endotoxin masking of different formulations within the downstream process of a biopharmaceutical product was analyzed. One out of eight different samples showed a strong masking capability in a hold-time study. The mechanism for endotoxin masking is reported to be dependent on the interplay between endotoxin, a chelator, and a non-ionic surfactant via a two-step mechanism. Contrary to this mechanism, masking of endotoxin was observed by a single substance, LDAO. Based on the results of the present study, masking of endotoxin by LDAO is proposed to follow a one-step mechanism according to the principles of the two-step mechanism. LDAO is used in various biopharmaceutical processes to inactivate different viruses; thus a suitable detection method for endotoxins in the presence of LDAO is of high interest for the pharmaceutical industry. First experiments on masking mitigation by the addition of dispersing agents and divalent cations did not increase endotoxin recoveries in LDAO samples. Further studies will be carried out to better understand the mechanism of LDAO-induced masking and subsequently how this effect could be overcome.

## Figures and Tables

**Table 1:** Matrix Composition of the Different Downstream Process Steps.

Step No.	In-Process Step	Components
1	Virus inactivation	Sodium, phosphate, chloride, tris, acetate, hydroxide, benzyl alcohol, kifunensine, MPA, LDAO, and protein
2	Affinity chromatography	Sodium, acetate, ammonium, sulfate, bis-tris, tris, chloride, citrate, benzyl alcohol, tris, and protein
3	pH adjustment	Sodium, acetate, ammonium, sulfate, bis-tris, tris, chloride, citrate, benzyl alcohol, and protein
4	Anion chromatography	Sodium, acetate, ammonium, sulfate, bis-tris, tris, chloride, citrate, and protein
5	Cation chromatography	Sodium, acetate, ammonium, sulfate, hydroxide, and protein
6	Virus filtration	Sodium, acetate, trehalose, hydroxide, and protein
7	Ultrafiltration/ diafiltration	Sodium, acetate, trehalose, polysorbate 20, and protein
8	Drug substance formulation	Sodium, acetate, trehalose, polysorbate 20, and protein
